# P135: Changing challenges into projects: a strategy to improve hand hygiene compliance rates

**DOI:** 10.1186/2047-2994-2-S1-P135

**Published:** 2013-06-20

**Authors:** E Tannous, B Hanan

**Affiliations:** 1 King Abdulaziz Medical City, Riyadh, Saudi Arabia

## Introduction

In 2007, King Abdulaziz Medical City (KAMC) was selected to be one of the WHO pilot testing sites to asses and evaluate the feasibility and acceptability of the recommendations expressed in the WHO draft guidelines on hand hygiene.

## Objectives

To develop a five years hand hygiene following the WHO project closure.

## Methods

A brainstorming session to highlight lessons learned from the testing phase and to identify challenges to changing Healthcare workers behavior and to develop strategy accordingly.

## Results

Challenges identified were the followings: 1- to involve all HCWs in the hand hygiene program, 2- to improve commitment through active participation in the decision making process, 3- to create an opportunity to network with each other and share knowledge, expertise and expected outcome towards the shared beliefs and values on the issue of safety, 4-To avoid the “one size fits all” strategy, 5- Creating a need for self improvement (for individuals) due to the team expectation, 6- to raise awareness of the risks to health when clean care is not attained and explain, in simple terms, to patients and their families, what are health care-associated infections and why they occur, 7- talk to patients and their families about hand hygiene and its role in the battle against health care-associated infections, which can be spread by hands, 8- SAVING LIVES.

New strategy was developed based on project management principles, the strategy was Changing Challenges into Projects, in simple words to have a project in each and every hospital unit/ward to tackle those identified challenges. The goal was to get to above, and maintain a 90% compliance rate; the scope, to be implemented in all clinical areas.

In order to eliminate misunderstanding of roles, the concept of program and project were described the hand hygiene program ownership was assigned to the Infection control department with a function to coaching and helping individuals developing internal and external structures that help them achieve success and to increase their potential by expanding their sense of what is possible. Projects were assigned to the champions whose role was to coordinate the projects at units level.

## Conclusion

This five year strategy was able to improve compliance rate at KAMC from 56% in 2008 to 86% in 2012.

## Disclosure of interest

None declared

